# Vat photopolymerization printing of functionalized hydrogels on commercial contact lenses

**DOI:** 10.1038/s41598-024-63846-7

**Published:** 2024-06-15

**Authors:** Muhammed Hisham, Haider Butt

**Affiliations:** Department of Mechanical and Nuclear Engineering, Khalifa University of Science and Technology, Abu Dhabi, 127788 UAE

**Keywords:** 3D printing, Vat photopolymerization, Contact lens, Colour blindness correction, UV monitoring, Polymers, Gels and hydrogels, Biomedical engineering

## Abstract

Contact lenses are widely used for vision correction and cosmetic purposes. Smart contact lenses offer further opportunities as functionalized non-invasive devices capable of simultaneous vision correction, real-time health monitoring and patient specific drug delivery. Herein, a low-cost vat photopolymerization technique is developed for directly 3D printing functionalized structures on commercially available contact lenses. The process enables controlled deposition of functionalized hydrogels, in customizable patterns, on the commercial contact lens surface with negligible optical losses. Multi-functional contact lenses can also be 3D printed with multiple materials deposited at different regions of the contact lens. Herein, the functionalities of colour blindness correction and real-time UV monitoring are demonstrated, by employing three suitable dyes incorporated into 2-hydroxyethyl methacrylate (HEMA) hydrogel structures printed on contact lenses. The results suggest that 3D printing can pave the way towards simple production of low-cost patient specific smart contact lenses.

## Introduction

Contact lenses are uniquely characterized by their low cost and widespread availability. It is estimated that over 150 million people use contact lenses worldwide^1^. Conventionally, contact lens usage has been limited to vision correction and cosmetic applications. However, the idea of smart contact lenses has gained traction recently, opening up a wide range of opportunities for contact lenses^2^. Smart contact lenses can serve as diagnostic and therapeutic devises for detection, control and treatment of various ocular illnesses^3,4^. In addition, their use as bionic contact lenses featuring virtual display units is also under exploration^5^. Recent investigations have already led to the development of smart contact lenses performing functions like intraocular pressure monitoring with concurrent drug delivery system for glaucoma control^4,6^, non-invasive diabetic monitoring^7,8^, and sustained personalized medication^9,10^. Contact lenses capable of continually monitoring eye movement^11^, ocular temperature^12^, tear pH^13^, various electrolytes^11^, and proteins^14^ have also been developed. Wireless charging and communication systems embedded in these contact lenses enable wireless operation and data transfer^4,8^. However, the production of these advanced contact lenses is highly challenging^15^. Complex production techniques lead to high production costs which would make smart contact lenses unaffordable for most of the population^16–18^.

Conventional contact lenses are mass produced through simple spin casting, lathe cutting and cast moulding techniques^19^. On the other hand, smart contact lenses require sophisticated chemical vapour deposition^3^, photolithography^4^ and etching^20^ techniques for its functional components. Laser ablation is also used to cut microchannels on the contact lens which are then selectively filled with suitable analytes^14^. Intricate components like electrodes, transistors and sensors are typically fabricated on suitable substrates and later transferred onto a normal contact lens^6,12^. The layer wise deposition and integration of these functional components is also challenging due to the material and geometrical limitations imposed by the contact lenses^12,21^. 3D printing enables controlled layer-by-layer deposition of materials in intricate three-dimensional patterns. 3D printing processes are also compatible with a variety of nanocomposite materials^22^. As a result, 3D printing of smart contact lenses is an attractive option. 3D printing can simplify the production process for smart contact lenses and enable their low-cost fabrication^19^. A few recent studies have explored the use of 3D printing for adding functionalized components on contact lens surfaces. Kong et al. demonstrated extrusion-based 3D printing of quantum dot light-emitting diodes (QD-LEDs) on a curved contact lens surface^21^. The contact lens was 3D scanned prior to the printing process to enable accurate material deposition on the curved geometry. Five different materials were printed in subsequent layers to form the QD-LEDs which displayed tuneable colour emission through electroluminescence^21^. Tam et al. used a novel nanoelectrospray 3D printing process to selectively deposit drug formulation on peripheral regions of a commercial contact lens^23^. The contact lens was placed on a custom-made lens holder and charged liquid droplets were selectively deposited through electric-field controlled spraying. Tetyczka et al. utilized inkjet printing to selectively print itraconazole nanocrystals on commercial 1-day contact lenses for direct drug delivery to the eye^24^. Pollard et al. printed glaucoma drug timolol maleate in ring patterns on commercial contact lenses through inkjet printing. In inkjet printing, the contact lenses are held in place by custom-made holders while drug-loaded ink is deposited onto it by the printer^25^.

Vat photopolymerization is one of the most commonly used 3D printing techniques. It is well-known as a low-cost high-resolution technique suitable for printing biomaterials like hydrogels^26^. Vat photopolymerization runs by selective crosslinking of liquid polymer resins through UV initiated crosslinking reaction. Repeated projection of UV light in subsequent layer-wise patterns produce solid 3D objects of required geometries^27^. Apart from biomaterials, vat photopolymerization can also print conductive materials, nanocomposites, and ceramics^28^. Vat photopolymerization printing is not affected by the viscosity or rheological properties of the print material. This flexibility in material properties is a huge advantage when compared to other 3D printing processes^29^. Processes like extrusion-based 3D printing, nanoelectrospray printing and inkjet printing, suffer severe limitations due to material viscosity^21,23–25^. Issues like nozzle clogging occur frequently in these printers causing print failures. Optimization of material viscosity and rheology is a daunting task for these printing processes^23–25^. Additionally, in vat photopolymerization there is good bonding between the deposited layers due to crosslink formation^26^. Extrusion printing and inkjet techniques do not always ensure sufficient bonding and may suffer issues of detachment of printed features^30,31^. Hence, vat photopolymerization holds great potential for easy production of stable functionalized components on contact lenses.

A few recent studies have explored the use of vat photopolymerization for 3D printing entire contact lenses^32–35^. However, 3D printers usually have difficulty printing thin curved structures like contact lenses. The issue of overcure occurs during the printing of thin curved structures^36,37^. Overcure results in unwanted extra curing which produces deviations from the intended geometry. At the same time, the layer-by-layer production process leads to staircase formation which reduces the surface quality^19,35^. Post-processing steps can be used to reduce the intensity of these issues. However, it is difficult to eliminate them completely when the entire contact lens is 3D printed^19^. On the other hand, 3D printing of thin curved structures is not required when the 3D printer is used to modify and functionalize a commercial contact lens. Here, the 3D printer is only used to deposit functionalized materials on a pre-existing contact lens which has the required curved geometry. The later process effectively eliminates several challenges associated with the 3D printing of contact lenses.

**Figure 1:**
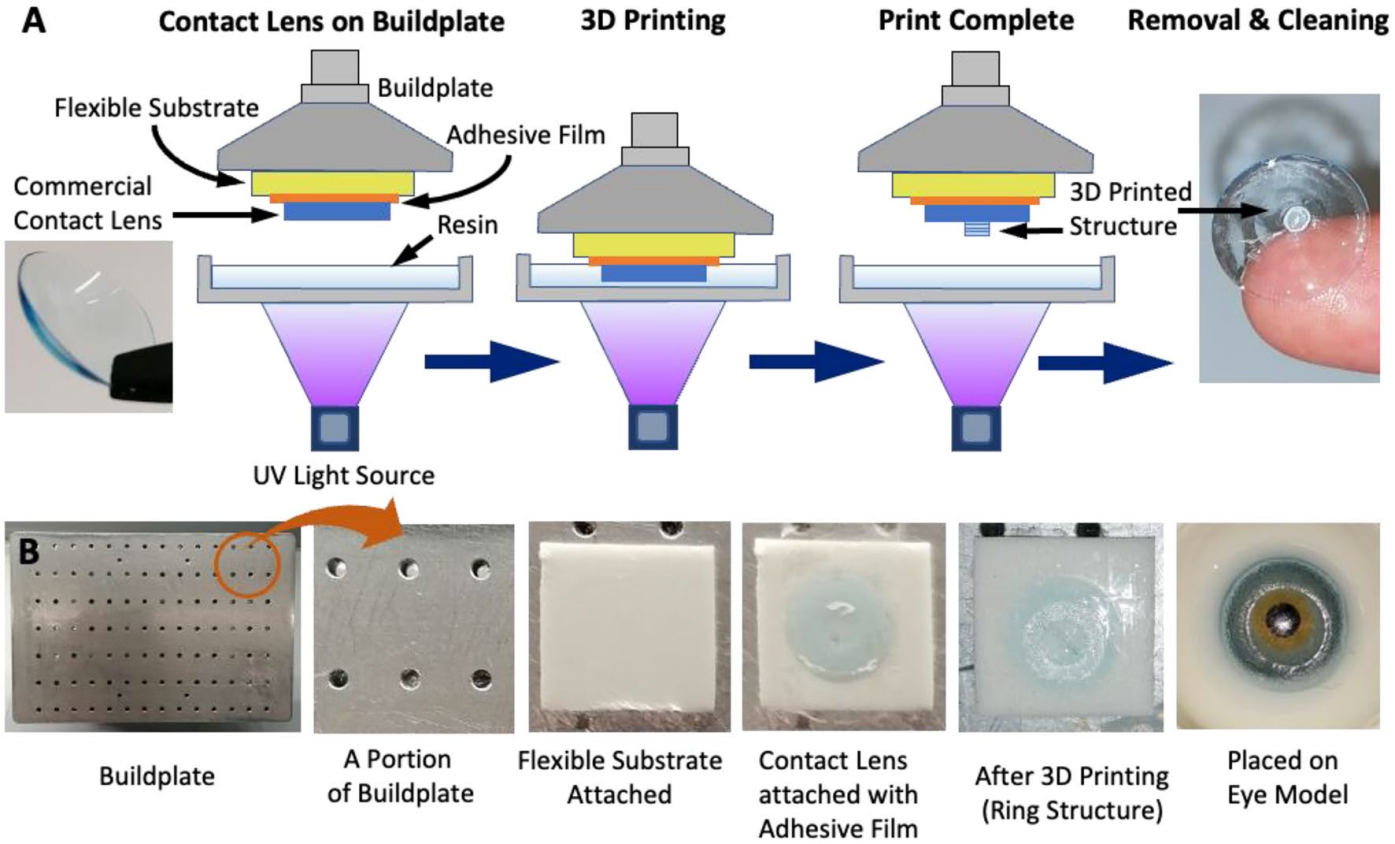
(**A**) Vat photopolymerization 3D printing on top of a commercial contact lens surface (attached to the buildplate). (**B**) Images describing the steps for attaching commercial contact lens on the printer buildplate.

Herein, we demonstrate the 3D printing of functionalized structures on commercial contact lenses using vat photopolymerization technique. The contact lens is first attached flat to the printer buildplate with a flexible substrate below it. The substrate provides a further smoothing effect for any remaining unevenness of shape. The contact lens is removed from the buildplate after the 3D printing process and regains its original curved geometry (Fig. 1A). The technique is compatible with commercial vat photopolymerization 3D printers, thus enabling easy low-cost 3D printing. Issues related to the curved geometry are overcome through the flat attachment of contact lens during printing process. Difficult pre-requisites like 3D scanning of the curved geometry are thus eliminated. Multiple materials can be 3D printed by repeating the printing process with different materials. The flexible substrate ensures that multiple print operations can be performed without affecting previously printed structures. Here, the technique is demonstrated for two different hydrogel combinations and for the functionalities of colour blindness correction and UV monitoring. 2-hydroxyethyl methacrylate (HEMA) based hydrogels were printed successfully on commercial contact lenses with excellent optical transmissivity. Functionalities of colour blindness correction and UV monitoring are demonstrated through suitable dyes added into the hydrogel. Atto565 and Atto488 dyes on the central region of the lens facilitate wavelength filtering for red-green and blue-yellow colour blindness correction. On the other hand, peripheral structures with UV sensitive photochromic dye aids smart real-time UV monitoring.

## Materials and methods

Wanhao Duplicator 8 was used for 3D printing. Wanhao Duplicator 8 uses a 405 nm UV LED along with LCD masking technology. Prior to 3D printing, the buildplate was modified by attaching a double-side flexible adhesive square to serve as flexible substrate for the contact lens (Fig. 1B). Above this substrate a small adhesive film of suitable adhesive strength was attached. An Acuvue® Oasys HydraLuxe™ 1-Day contact lens was then attached flat on this film. The lens was placed on a finger and directly pasted on the adhesive film on the substrate, as shown in supporting information (Fig. S1). The substrate and adhesive film facilitate easy flat attachment of the contact lens without any wrinkles. The 3D printer was re-calibrated to account for the buildplate modification. A CAD model was then prepared on Solidworks, sliced using Chitubox slicer and transferred to the 3D printer. The alignment of the image projection and the contact lens was confirmed by ensuring that the image is projected at the required location in the vat. Liquid resin was then poured into the vat and 3D printing was performed. After the printing process, the contact lens was carefully removed and washed in contact lens solution (ReNu multipurpose solution, Bausch & Lomb). 2-hydroxyethyl methacrylate (HEMA), polyethylene glycol diacrylate (PEGDA), and de-ionized water were mixed in suitable ratios to obtain the liquid resin for 3D printing. Two different volumetric ratios were used in this study: HEMA:PEGDA 50:50 (with no water), and HEMA:PEGDA:Water 72:3:25. Trimethyl benzoyl diphenylphosphine oxide (TPO) was added as photoinitiator to this mixture at 5 wt.%. The mixture was stirred for 20 min using a magnetic stirrer. Atto565, Atto488 and photochromic dye were then added to this mixture before 3D printing. The equilibrium water content was measured by drying the 3D printed hydrogel samples and then immersing them in distilled water. The weight of the sample was measured at different time intervals and used to calculate the water content. 3D printed hydrogel discs of 300 μm thickness were used for this test. The following equation gives the equilibrium water content:

1 $$Equilibrium\, Water\, Content \left(\%\right)=\frac{Final\, weight-Dry\, weight}{Final\, weight}$$

Contact angle was measured by sessile drop method^38^. A 3 μl DI water droplet was dropped on the sample and the droplet shape was imaged. ImageJ plugin was then used to calculate the contact angle from this image. ImageJ was also utilized to measure the radius of curvature of the contact lens before and after 3D printing.

## Results and discussion

The 3D printing process enabled the production of contact lenses with well-defined hydrogel structures on its surface. Required features could be added to contact lens without affecting its curved geometry. Although this study used Acuvue® Oasys HydraLuxe™ contact lenses, the same process can be replicated with most other commercial contact lenses. Commercial contact lenses have ultra-thin (typically 100–200 µm) soft flexible structure. This soft flexible nature enables their reversible deformation to a planar shape. However, a slight unevenness is often apparent between the central region and the peripheries while in the planar shape. Herein, the substrate provided below the contact lens ensured that this unevenness does not affect the 3D printing process. The substrate changes shape due to compressive forces between the vat and buildplate, ensuring a flat surface for the printing process. The soft ultra-thin nature of commercial contact lenses however poses difficulties during de-attachment. The use of strong adhesives for attachment to the buildplate results in the contact lens tearing during removal. On the other hand, weak adhesives cause the contact lens to fall off from the buildplate during printing process. Improper adhesion also causes the liquid resin to reach beneath the contact lens forming undesired crosslinked structures there. Thus, the selection of an adhesive film with optimal adhesion level is of major importance in this printing technique. Herein, the optimal adhesive film was chosen by trial and error, considering various types of films. The alignment of image projection with the contact lens is also slightly challenging. In this work, the alignment was done manually for each sample (Fig. S2). However, the alignment can also be done by the addition of a permanent structure on the buildplate which ensures that the contact lens is always at a well-aligned fixed position (Fig. S3). Such buildplate modifications will facilitate easy alignment and eliminate the need for repeated alignment for each sample. After 3D printing, the contact lens was stored in commercial contact lens solution (ReNu multipurpose solution, Bausch & Lomb) to maintain full hydration. When stored properly, the modified contact lens was found to last for very long periods without significant damage (Fig. S4).

**Figure 2:**
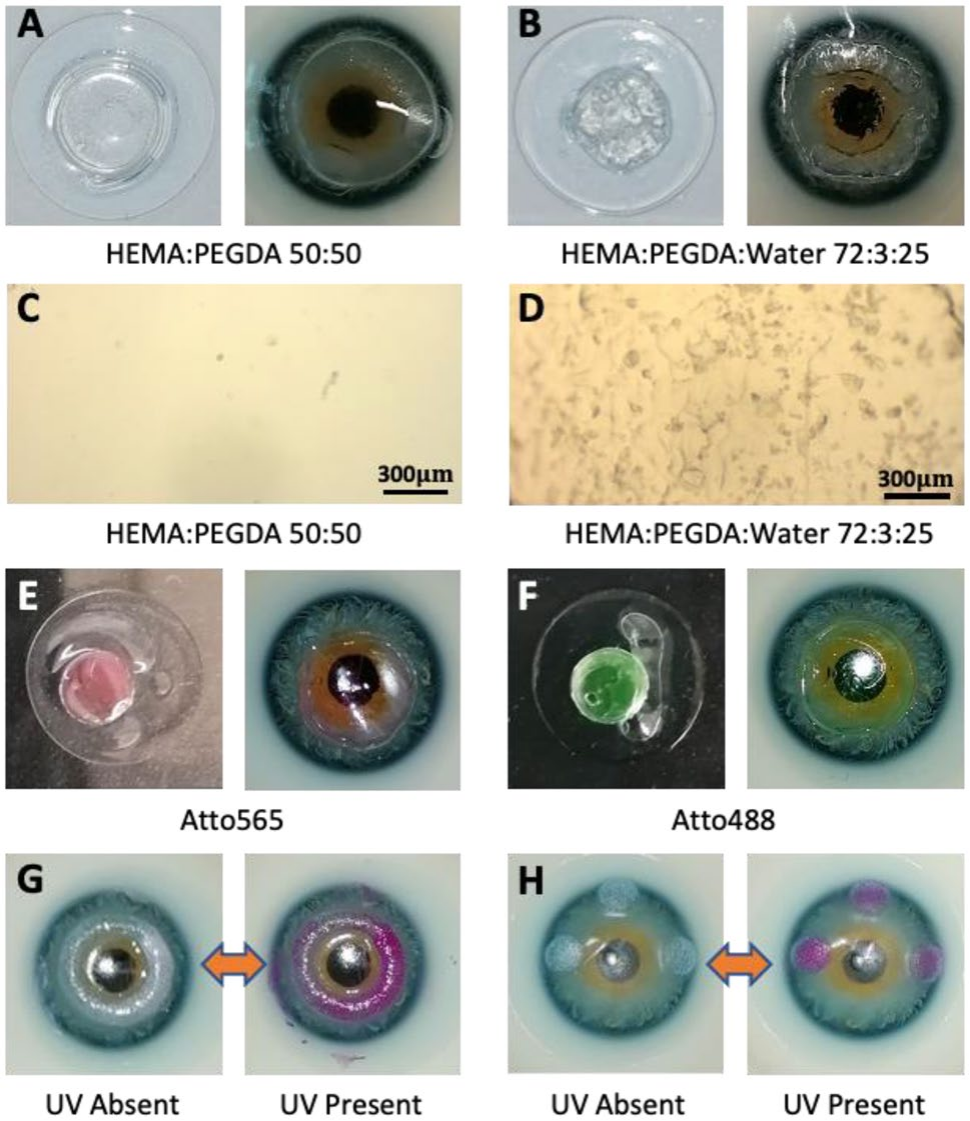
(**A**, **B**) 3D printed disks on commercial contact lens. (**C**, **D**) Magnified surface optical microscopy images of the printed structures. (**E**, **F**) Atto565 and Atto488 disks printed on contact lens for colour blindness correction application. (**G**, **H**) Photochromic ring and disk structures on commercial contact lens for UV monitoring.

3D printed structures with two HEMA based hydrogels are shown in Fig. 2A,B. HEMA:PEGDA 50:50 was found to produce features with better resolution. However, HEMA:PEGDA:Water 72:3:25 had material properties closer to the contact lens material due to its higher water content. The latter combination also had a softer nature which matched well with the commercial contact lens. The addition of large amount of water however affected the print resolution and surface quality of the printed hydrogel. The surface of HEMA:PEGDA:Water 72:3:25 had pores which slightly reduces the optical transmission and produces a rougher surface. On the other hand, HEMA:PEGDA 50:50 had much better surface quality without any pore formation (Fig. 2C,D). Colour blindness correction dyes Atto565 and Atto488 were added to HEMA:PEGDA 50:50 at 0.005 wt.% concentration and 3D printed as central disks on the contact lens (Fig. 2E,F). These two dyes correct red-green and blue-yellow colour blindness respectively^39,40^. Photochromic rings were 3D printed by adding 30 wt.% of UV sensitive photochromic dye to HEMA:PEGDA:Water 72:3:25 formulation and printing centred rings on the contact lens (Fig. 2G). These rings enable UV monitoring without affecting the vision. The ring reversibly changes colour from white to pink in presence of UV radiation. Photochromic structures were also printed as small disks around the centre of the contact lens (Fig. 2H). Photochromic structures can be integrated with smart contact lens monitoring systems to monitor instances of hazardous UV exposure to the eye.

**Figure 3:**
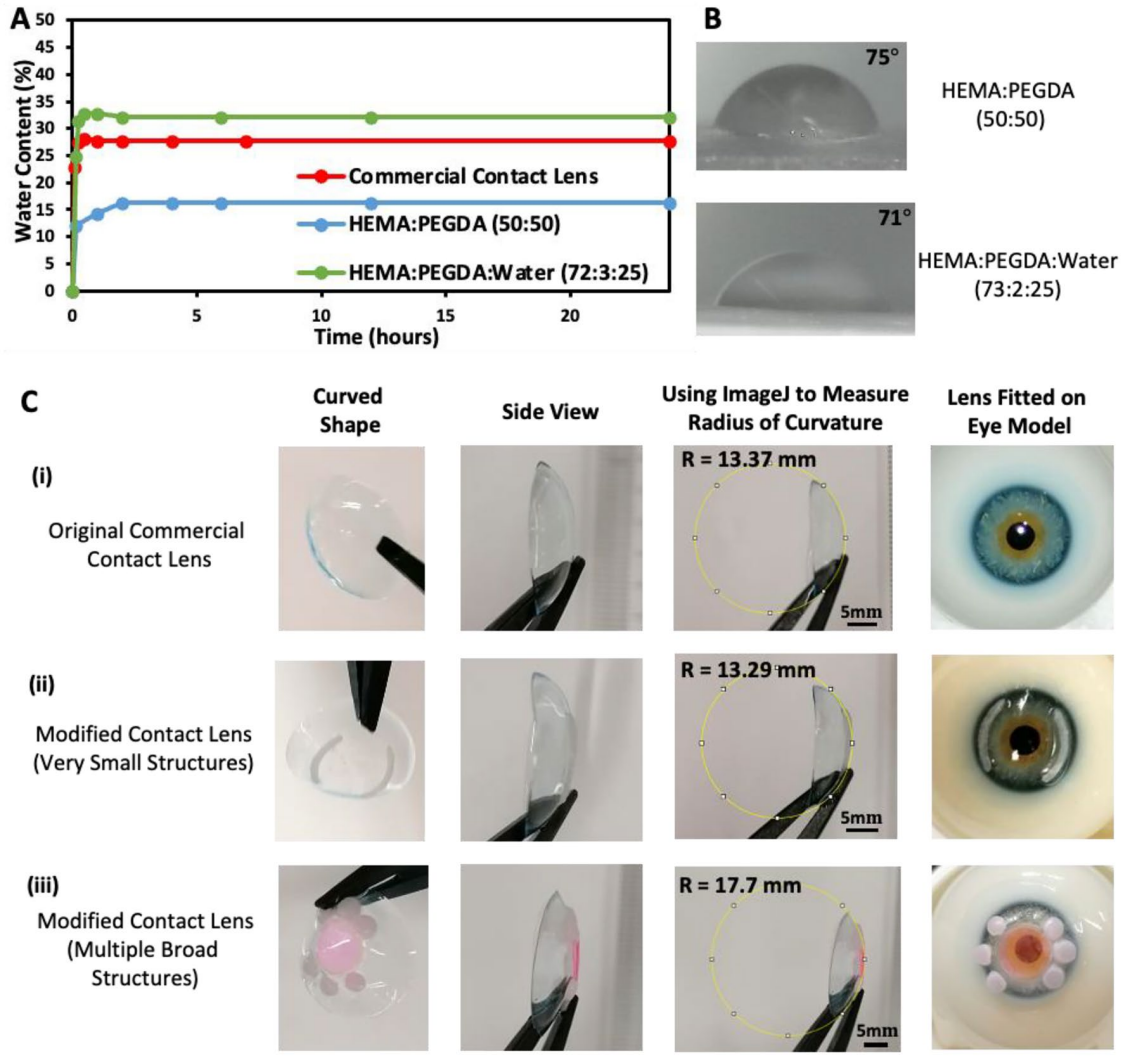
(**A**) Water absorption and (**B**) contact angle of the two 3D printed hydrogels combinations. (**C**) The shape and radius of curvature measurement of the contact lens before and after the 3D printing process.

The material properties of HEM:PEGDA 50:50 has been was evaluated in a previous work^32^. This hydrogel combination has properties closer to rigid contact lenses which have high tensile modulus, low water content and low oxygen permeability. The equilibrium water content was measured here for this combination as 16% (Fig. 3A). However, as these structures are printed only on a limited portion of the commercial contact lens, the remaining regions would retain their original optimal properties (i.e., high water content and high oxygen permeability). HEMA:PEGDA:Water 72:3:25 had a higher water content of 32% (Fig. 3A). However, lower optical quality and surface quality is again a disadvantage for this combination. For optimal properties, water addition before 3D printing can be possibly avoided and HEMA concentration can be increased. 3D printed hydrogels with high HEMA content and no water addition prior to 3D printing displayed high equilibrium water content and a tensile behaviour that matches soft contact lens requirements, while retaining good surface quality (Fig. S5). All hydrogel combinations had suitable contact angle below 90° (Fig. 3B). A low contact angle below 90° ensures a hydrophilic surface with suitable wettability.

After 3D printing, the contact lenses were washed in commercial contact lens solution and sonicated for a few minutes to remove any traces of impurities from the contact lens. The washed lenses were inspected visually and by checking the optical transmission of the original commercial lens region. The lenses were found to be free from impurities and retains its original properties. Other suitable liquids can also be used for better cleaning, as long as they do not damage the lens. Commercial contact lenses are usually designed to sustain some amount of UV exposure without damage. Hence, the brief UV exposure in the 3D printer is not expected to damage the lens. Several commercial contact lenses now have UV blocking characteristics, which again implies that they can sustain some UV exposure without damage. The Acuvue® Oasys HydraLuxe™ that we used in this study had Class-1 UV blocking characteristics. The shape of the contact lens before and after 3D printing was evaluated by imaging it sideways and using ImageJ to measure the curvature (Fig. 3C). The lenses were held by a tweezer at one end and held sideways for this measurement. All contact lenses were imaged in the exact same manner to enable a comparison between them. For very small 3D printed structures, there was no significant deviation in the curvature of the contact lens (Fig. 3Ci, ii). Broader structures covering large area on the lens did produce a deviation in curvature (Fig. 3Ciii). However, the modified contact lenses were still found to fit on an eye model, which indicates that lens may still be suitable for actual use. Due to its soft flexible nature, contact lenses can accommodate some changes in dimensions when placed on the cornea. In Fig. 3C, the measured radius of curvature differs from the base curve (BC) of the commercial lens, which is reported as 8.5 mm by the manufacturer. This difference is thought to be due to the effect of gravity and the tweezer used to hold the lens during measurement. However, as the exact same procedure is followed for all lenses, the above measure serves the purpose of a means of shape comparison between the lenses. The above two tests (radius of curvature measurement and the fitting on eye model) when taken together indicates that broad structures do produce some deviation in shape, but the deviation is still minor as the lens still fits on an eye model. Future studies can focus on better measurement techniques for accurate measurement of the BC of modified lenses and actual patient trials to further confirm the wearability of these lenses. The thickness of 3D printed structures was evaluated by imaging the cross-section of the modified lens under an optical microscope (Fig. S6). As we used an ultra-low-cost 3D printer, the printer resolution posed limitations in the minimum thickness of printed structures. However, structures of thicknesses as low as 130 μm could be 3D printed despite the challenges (Fig. S6). 3D printers with better resolution can be employed in future studies for printing structures of lower thicknesses. 3D printers capable of printing in sub-micrometre range (such as two-photon polymerization 3D printers) are currently available. Such printers can be utilized for high resolution patterns which produce minimal changes in lens thickness. The 3D printing of thinner structures with material properties that perfectly match the properties of the original commercial contact lens may also help in eliminating potential deviations in curvature.

**Figure 4:**
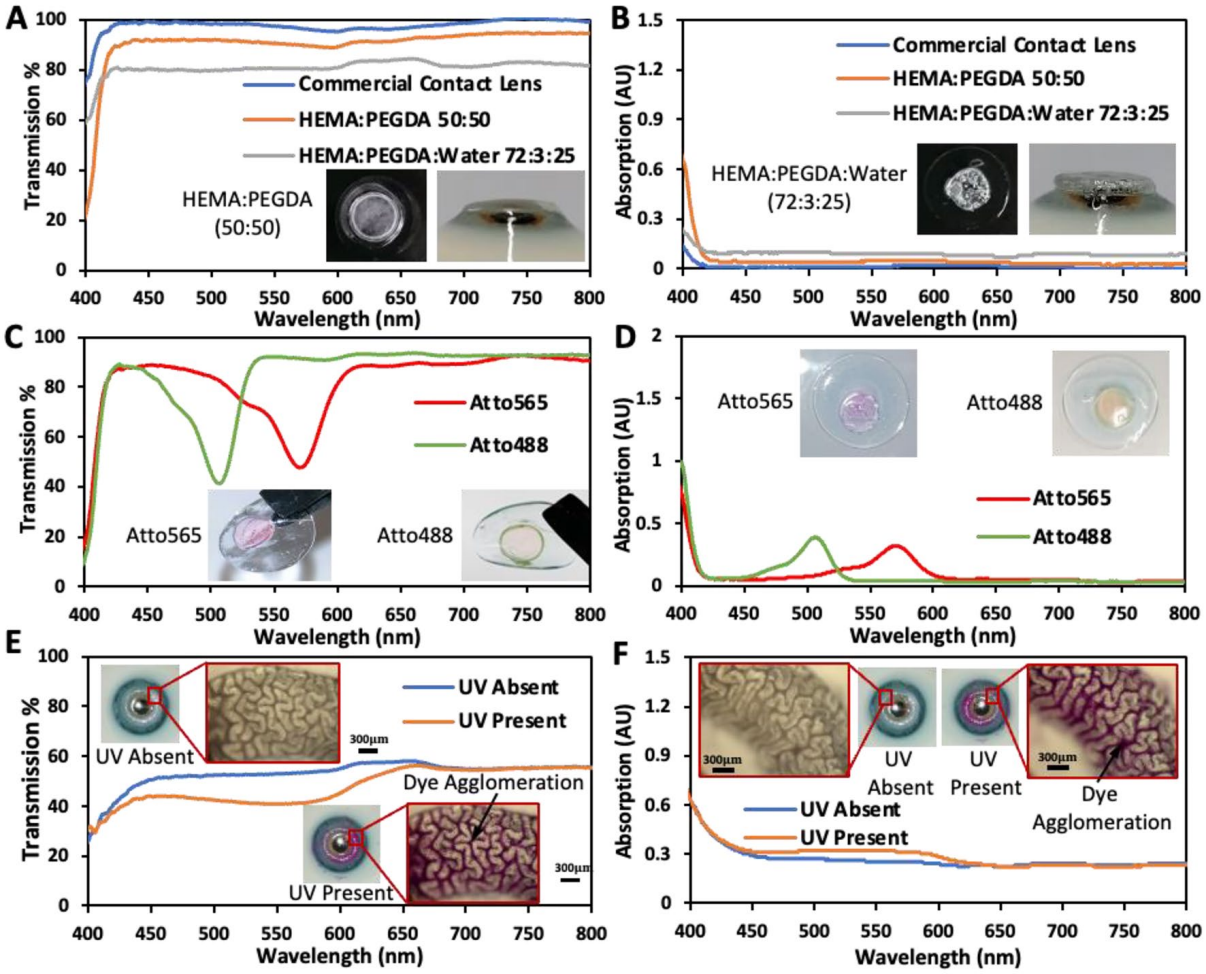
(**A**, **B**) Transmission and absorption spectra from hydrogel disks 3D printed on contact lens. (**C**, **D**) Transmission and absorption spectra from Atto565 and Atto488 disks printed on contact lens. Inset shows the printed features on contact lenses. (**E**, **F**) Transmission and absorption spectra from photochromic structure 3D printed on contact lens. Inset shows magnified images of photochromic rings. The transmission measurements were done in fully hydrated state with at ambient relative humidity of 65%.

Optical transmission and absorption measurements were obtained with Ocean Optics UV–vis spectrophotometer (USB 2000 + , by Ocean Optics) with 200–1100 nm detection range. The two hydrogels both displayed good optical properties when 3D printed onto commercial contact lenses (Fig. 4A,B). Commercial contact lens itself had an average transmission of 98%. HEMA:PEGDA 50:50 disks printed on the contact lens also had excellent optical transmission, retaining a transmission above 90%. HEMA:PEGDA:Water 72:3:25 had transmission above 80%. The latter hydrogel maintains an acceptable transmission level despite issues of pore formation. Contact lenses with Atto565 and Atto488 disks displayed selective wavelength filtration facilitating colour blindness correction (Fig. 4C,D). Red-green colour blindness correction is enabled by filtering 520–580 nm wavelengths^41^. Whereas, blue-yellow colour blindness correction is facilitated by filtering 440–500 nm wavelengths. 3D printed Atto565 contact lenses had a transmission dip and which peaks at 570 nm with a full width at half maxima (FWHM) 545–590 nm. Atto488 contact lenses had a similar behaviour, with the exception that peak was at 505 nm with FWHM 485–520 nm. These two contact lenses would be suitable for patients suffering from red-green and blue-yellow colour blindness, respectively. The level of wavelength filtration can be varied as per patient needs by adjusting the concentration of the dye in hydrogel fomulation. The spectra of 3D printed photochromic rings show significant variation in presence/absence of UV radiation (Fig. 4E,F). A drop in optical transmission occurs in the presence of UV in the range 410–670 nm, with the largest variation occurring at 570 nm (12% drop). Microscope images show the agglomeration of photochromic dye in the printed rings due to very high dye concentration (30 wt.%).

**Figure 5:**
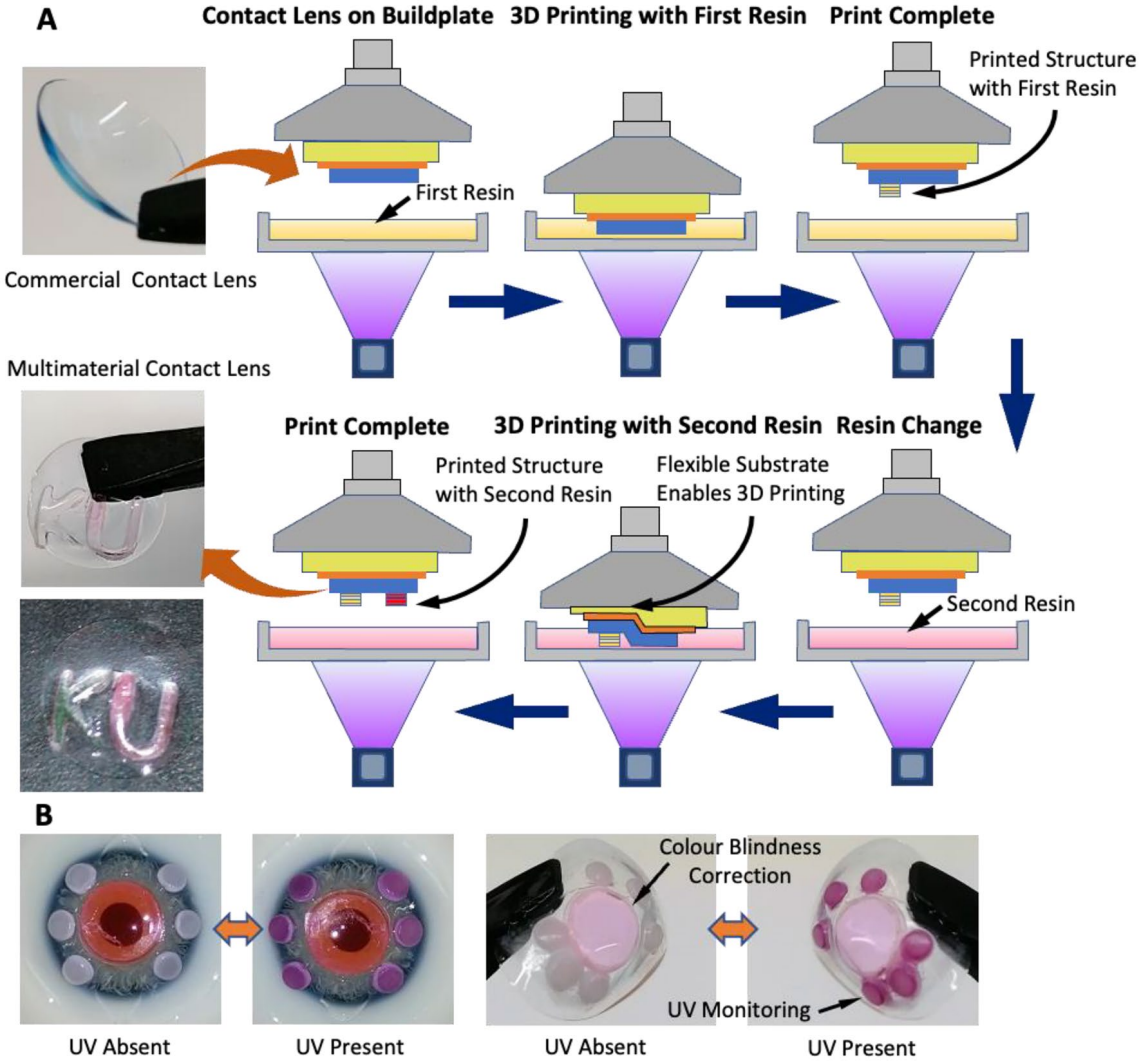
(**A**) The process of 3D printing multimaterial structures on commercial contact lenses. (**B**) Multi-functional contact lenses with colour blindness correction (Atto565 dye) and UV monitoring functionalities.

Functionalized components composed of multiple materials can be 3D printed on commercial contact lenses by repeating the printing process with each material. Figure 5A illustrates the process of 3D printing structures with multiple materials. The flexible substrate beneath the contact lens enables subsequent printing of multiple materials. After the first 3D printing step, structures of the first material are present on the surface of the contact lens. Now, if 3D printing is done without a flexible substrate, these printed structures will hit the bottom of the vat and get damaged. The flexible substrate protects these structures from damage by its selective deformation (Fig. 5A). Previously printed structures get pushed inwards due to compressive forces from the vat, the substrate underneath them getting compressed. The other regions do not get pushed inwards and hence these regions devoid of printed structures are exposed and brought outwards for 3D printing. Multi-functional contact lenses can be easily produced using this process. Multi-functional contact lenses with concurrent colour blindness correction and UV monitoring functionalities were produced as proof of concept (Fig. 5B).

The above 3D printing process can be utilized with suitable diagnostic and therapeutic materials to produce smart multi-functional contact lenses with real-time disease monitoring and stimuli-responsive drug delivery. Future studies can focus on the actual production of such diagnostic/therapeutic contact lenses through vat photopolymerization technique. Drug loaded structures can be 3D printed on the contact lens surface and can provide sustained drug delivery to the eye. The drug type, dosing and release rate can be adjusted according to patient needs. Suitable dyes or nanomaterial structures 3D printed on the contact lens can be utilized for ocular health monitoring from the contact lens. Parameters like glucose level in tears, tear pH, ocular temperature, and the levels of certain proteins and biomarkers can be monitored using functionalized contact lens with 3D printed structures. Such advanced lenses can facilitate early detection and control of several diseases. The use of two-photon polymerization (TPP) for 3D printing on contact lenses is another important field for future exploration. Vat photopolymerization printers employing TPP technology can produce structures with sub-micrometre thicknesses with high resolution. 3D printing of such ultra-thin structures will provide better wearer comfort and eliminate any potential deviation in the lens structure. TPP with suitable conductive resins can easily produce smart contact lenses with complex electronic networks and advanced sensing capabilities. Future studies can also focus on further material optimization and patient trials for these modified contact lenses. We envision that further developments in vat photopolymerization 3D printing will enable easy low-cost smart contact lens production.

## Conclusions

A vat photopolymerization technique was successfully demonstrated for 3D printing functionalized components on commercial contact lenses. Multi-functional contact lenses with simultaneous colour blindness correction and UV monitoring functionalities were 3D printed with this technique. The technique is characterized by ease of operation and low-cost when compared to other smart contact lens production processes. Hydrogel composition plays a major role in the print resolution and optical characteristics of printed structures. HEMA:PEGDA (50:50 ratio) formulation offers excellent print resolution, surface quality and 90% optical transmission when printed on contact lenses. Atto dyes printed on contact lens provides suitable wavelength filtering for colour blindness correction, while photochromic features display reversible UV-initiated colour change. Vat photopolymerization 3D printing offers unique opportunities for smart contact production. Effective utilization of vat photopolymerization and further optimization of print parameter can aid in fabrication of affordable personalized smart contact lenses.

### Supplementary Information

Supplementary Information.

## Data Availability

The datasets used and/or analysed during the current study available from the corresponding author on reasonable request.
