# Supplementary material for: Vat photopolymerization printing of functionalized hydrogels on commercial contact lenses
Source: Sci Rep. 2024 Jun 15;14:13860. doi: 10.1038/s41598-024-63846-7 (PMC11180191; doi:10.1038/s41598-024-63846-7)
Supplement: Supplementary file 1 — Supplementary Information. [file 41598_2024_63846_MOESM1_ESM.docx]

**Supporting Information**

**Vat Photopolymerization Printing of Functionalized Hydrogels on Commercial Contact Lenses**

Muhammed Hisham^a*^, Haider Butt^a*^

^a^ Department of Mechanical & Nuclear Engineering, Khalifa University, Abu Dhabi, 127788, UAE

**Corresponding Author:* [*100060533@ku.ac.ae*](mailto:100060533@ku.ac.ae) *(Muhammed Hisham),* [*haider.butt@ku.ac.ae*](mailto:haider.butt@ku.ac.ae) *(Haider Butt)*

**No. of Pages: 7**

**No. of Figures: 6**


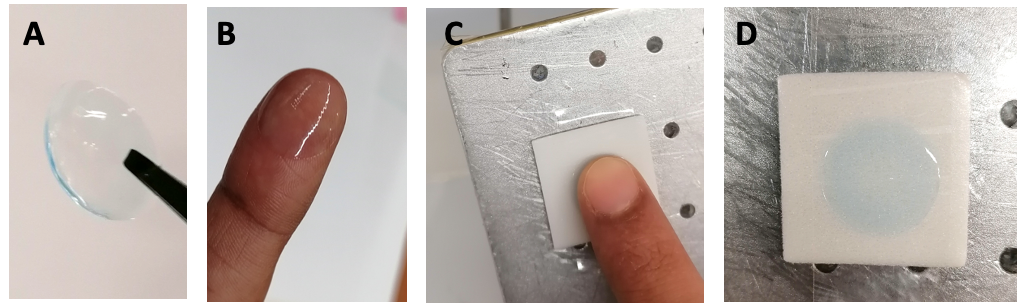


**Figure S1**. Attaching the contact lens on the flexible substrate. (A) A commercial contact lens. (B) The lens is placed on fingertip. (C) Pasting the lens on the substrate. (D) The contact lens attached flat on the substrate does not have any wrinkles.

**3D Printer Alignment – Manual Method**

In manual alignment, first the exact spot on the vat corresponding to the attached contact lens is determined. This is done by identifying the spot manually, as shown in **Figure S2 A**. The required spot may also be identified by moving down the buildplate until it presses firmly on the vat, which often leaves a mark on the vat showing the boundaries of the contact lens. Once the required spot is identified, the points corresponding to the centre and boundaries of the contact lens are marked by suitable identifiers (**Figure S2 B**). Next, the required CAD model is opened in a slicing software, aligned close to the required spot as possible(**Figure S2 C**). The file is then uploaded to the 3D printer. The printed is started with the buildplate paused. The location of UV projection on the vat is checked. The position of CAD model in slicing software is changed and above step repeated, until the UV projection coincides with the previously marked spot on the vat (**Figure S2 D,E**). Now, the alignment is complete. The 3D printed structure will now exactly fall on the attached contact lens.


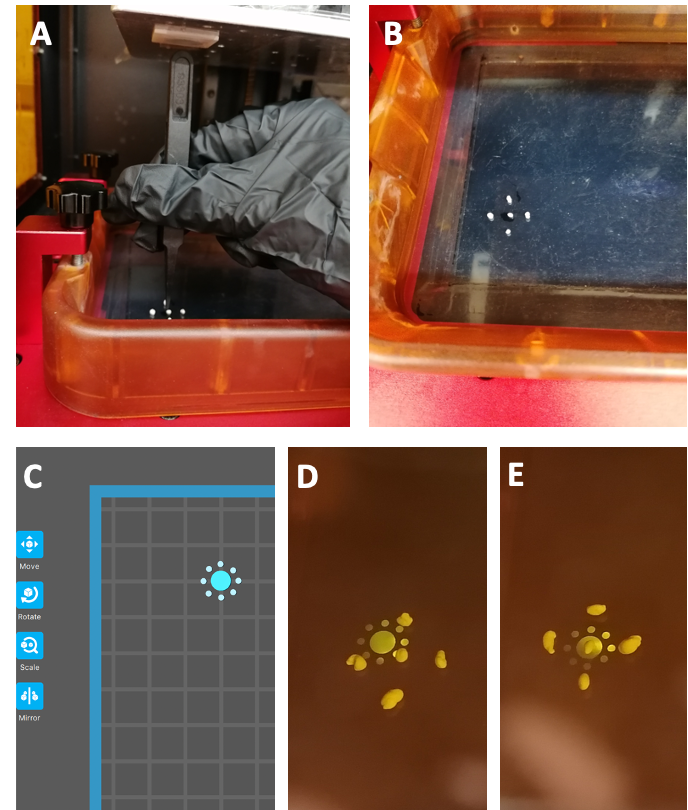


**Figure S2.** Method of manual alignment used in this work. (A) manually identifying the required spot on the vat. (B) The required spot is marked with small markers. (C) The required CAD file is placed in slicing software as close as possible to the required spot. (D) The UV projection slightly deviated from the required spot. (E) A well aligned UV projection matching with the centre of the required spot after correcting the positioning in slicing software.

**3D Printer Alignment – Permanent Alignment**

In the permanent alignment (which is suitable for long term use), the buildplate is modified by adding a structure which has the exact diameter of a flat contact lens. The structure itself can be 3D printed on the buildplate with a durable material and fixed firmly with suitable adhesives at its base (**Figure S3 A,B**). The location corresponding to this structure on the slicing software (X and Y coordinates) is identified and noted (**Figure S3 A**). For 3D printing, the flexible substrate and the contact lens is attached to the modified structure on the buildplate (**Figure S3 C**). The 3D printer is then re-calibrated by standard printer calibration procedure to account for the buildplate modification. For printing, the CAD model to be 3D printed can be placed within the XY coordinates previously determined on the slicing software (**Figure S3 D**). The CAD model will then be exactly 3D printed on the attached contact lens.


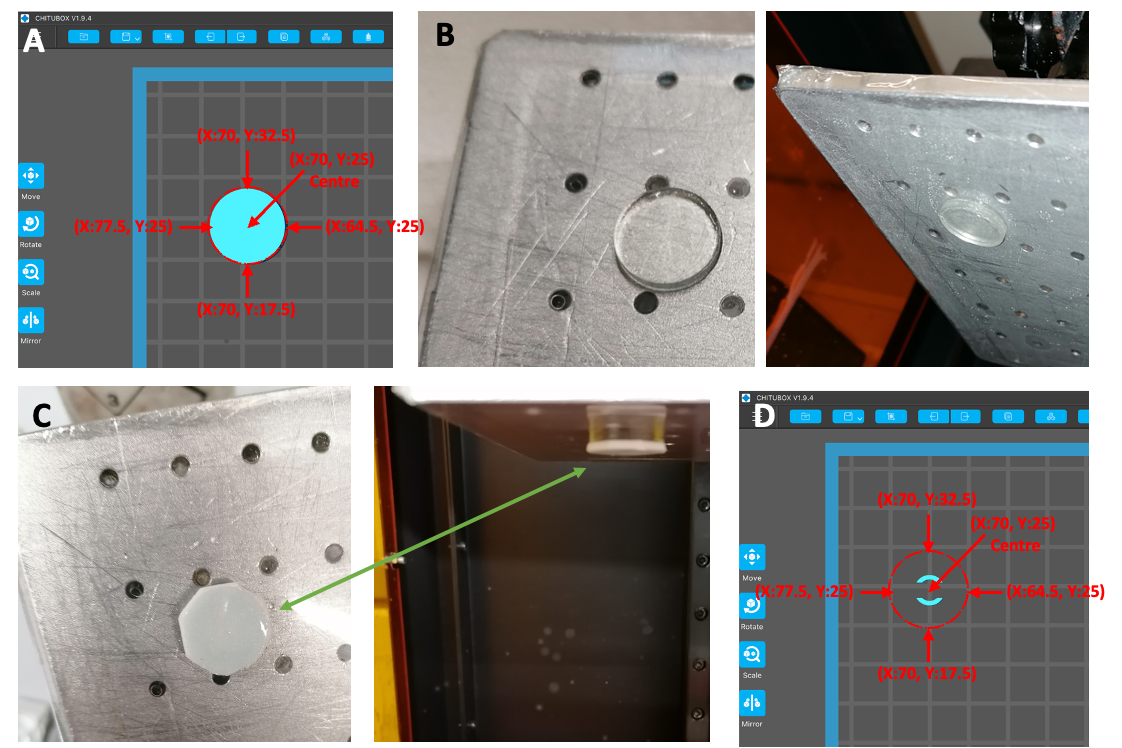


**Figure S3.** Buildplate modification for permanent alignment. (A) A structure for buildplate modification in slicing software with coordinates identified. The structure has dimensions that match a commercial contact lens. (B) The modified buildplate. (C) Flexible substrate and contact lens attached on the modified buildplate. (D) The CAD models to be 3D printed on the contact lens are placed within the identified coordinates in slicing software. The model will then be 3D printed exactly on the attached contact lens.


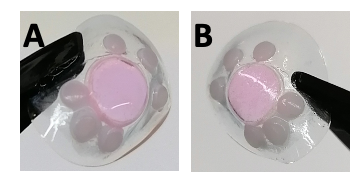


**Figure S4.** The modified contact lens: (A) soon after production, and (B) after a period of three months.


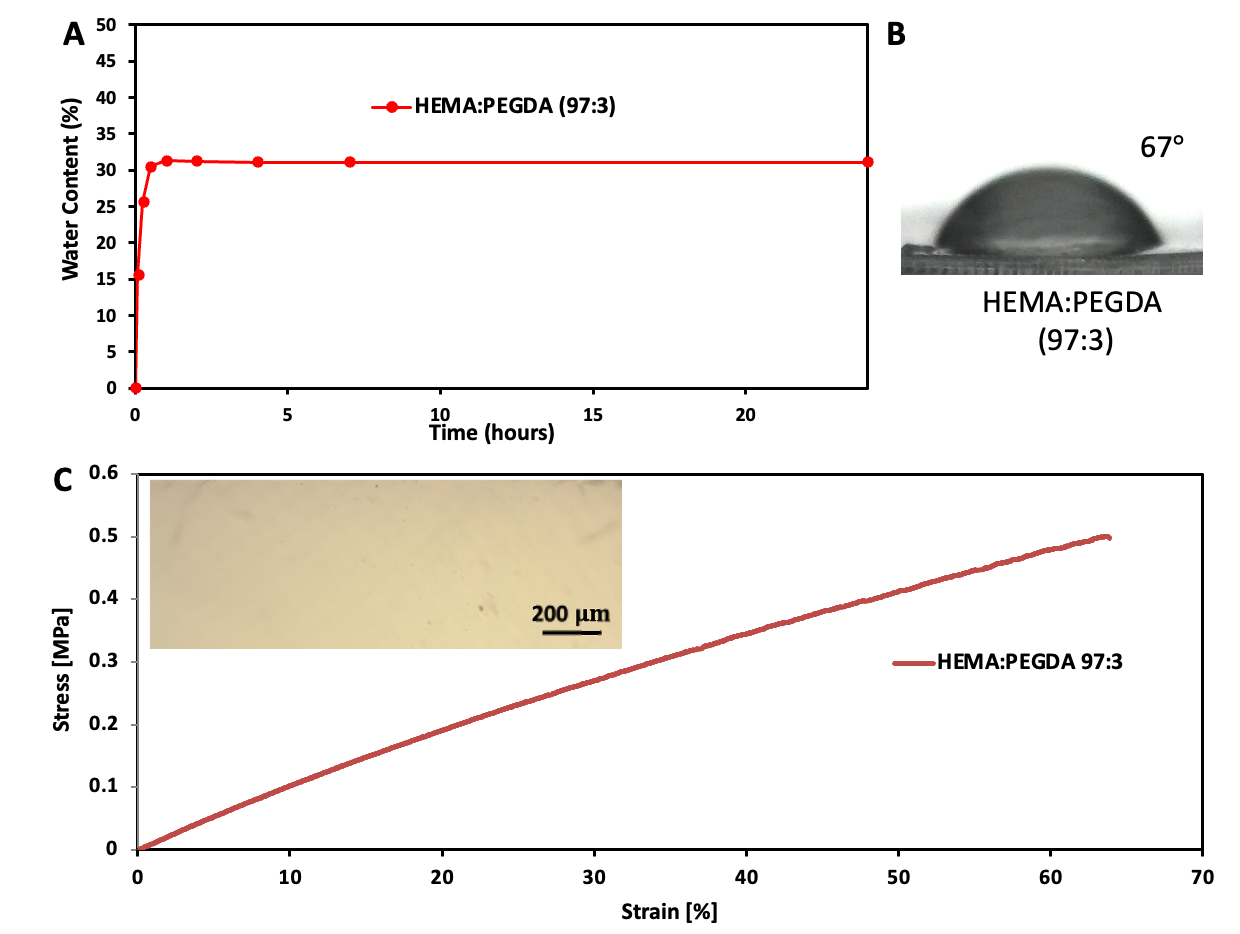


**Figure S5**. (A) Water absorption, (B) contact angle and (C) Tensile behaviour 3D printed HEMA:PEGDA 97:3. The tensile modulus was tested on 3D printed ASTM D638 Type IV samples. The tensile test was performed on Zwick-Roell Z005 UTM (2.5 kN load cell) at a test speed of 1mm/min. Tensile modulus determined as 1.05 MPa, which exactly falls in the range of commercial soft contact lens materials. Inset in (C) shows surface image the 3D printed HEMA:PEGDA 97:3 sample showing good surface quality.

**
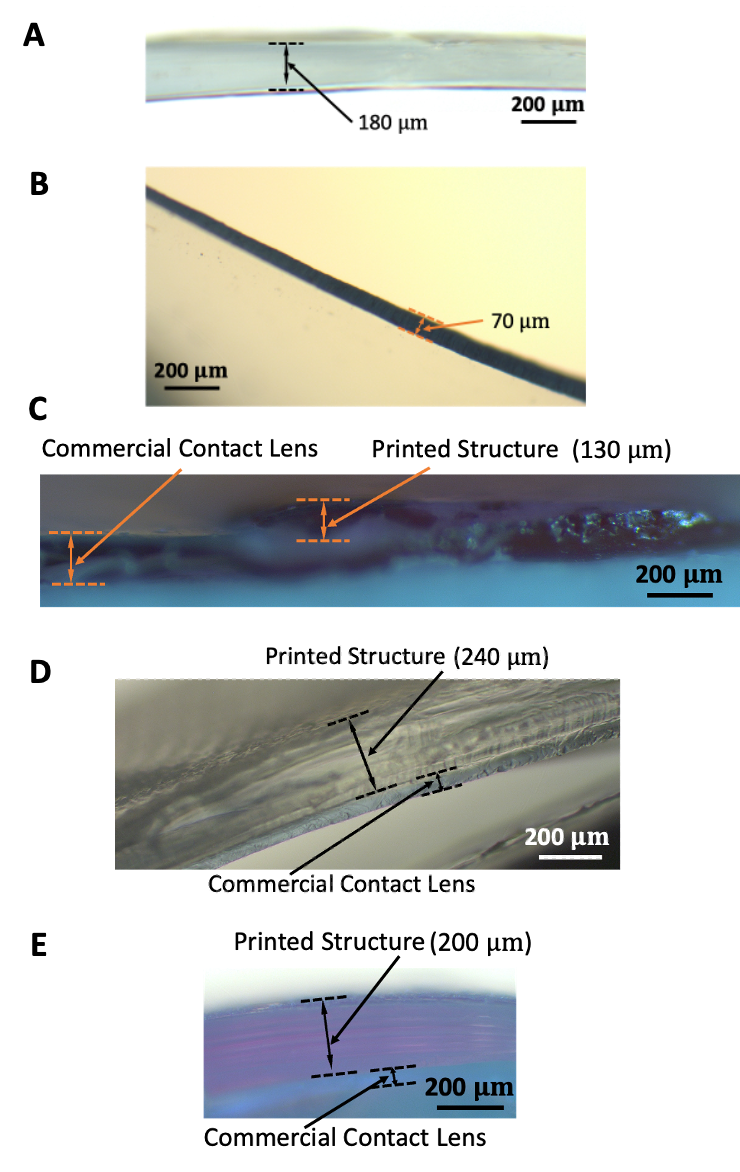
**

**Figure S6.** Optical microscope images of cross-sections of commercial contact lens and modified contact lens. (A) Edge of commercial contact lens. (B) Centre of commercial contact lens. The commercial lens has a thickness around 180 $\mu m$ at the edges and a thickness below 100 $\mu m$ at the centre. (B) Contact lens with thin printed structure (thickness of structure: 130 $\mu m$). (C) Contact lens with printed structure of thickness 240 $\mu m.$ (D) Contact lens with printed structure of 200 $\mu m$ thickness.
